# Changes in serum sodium during an external ventricular drain weaning in patients with subarachnoid hemorrhage

**DOI:** 10.3389/fneur.2026.1843156

**Published:** 2026-08-19

**Authors:** Paul Horton, Yajun Mei, Chelsea Ponder, Lisa Danyluk, Kate Martin, Feras Akbik, Owen B. Samuels, Ofer Sadan

**Affiliations:** 1 H. Milton Stewart School of Industrial and Systems Engineering, Georgia Institute of Technology, Atlanta, GA, United States; 2 Department of Biostatistics, School of Global Public Health, New York University, New York, NY, United States; 3 Division of Neurocritical Care, Department of Neurology and Neurosurgery, School of Medicine, Emory University, Atlanta, GA, United States

**Keywords:** external ventricular drain, obstructive hydrocephalus, serum sodium, subarachnoid hemorrhage, ventriculoperitoneal shunt

## Abstract

**Background:**

Dysnatremia is a common complication following non-traumatic subarachnoid hemorrhage (SAH). Potential causes include induced hypernatremia and hyponatremia related to cerebral vasospasm or cerebral salt wasting syndrome. Based on bedside observations, this study is aimed to investigate the correlation between changes in serum sodium during an external ventricular drain (EVD) wean trial and wean trial outcomes.

**Methods:**

A retrospective analysis was performed on a cohort of patients admitted with SAH from a large volume tertiary center. The cohort comprises patients admitted to the neuroscience ICU between 2012 and 2022 with subarachnoid hemorrhage complicated by obstructive hydrocephalus. The primary outcome was defined as successful wean if the EVD was removed at the end of a wean attempt or failed if the EVD was reopened or if a ventriculoperitoneal shunt was placed.

**Results:**

The cohort included 985 patients and consisted of 1,318 unique wean attempts, of which 790 were successful and 528 were unsuccessful. Patients with failed attempts had overall higher clinical and radiographic hemorrhage grades. Serum sodium concentration (mEq/L, mean ± standard deviation) on the day of wean initiation was 139.3 ± 8.5 in the successful wean group versus 140.7 ± 6.6 in the failed group (*p* < 0.001). During the first 3 days of the wean attempt, serum sodium decreased by 0.8 ± 7.8 mEq/L in the successful group compared with 1.5 ± 5.8 mEq/L in the failed group (*p* = 0.048). There was no statistically significant difference in the cumulative sodium intake before (up to 2 days) or during the wean attempt between the groups. In a multivariable mixed effect logistic regression analysis, the slope of the change in serum was associated with the wean attempt outcome (odds ratio [95% confidence interval] of 1.324 [1.174–1.506]). When cases with positive or negative sodium changes were analyzed separately, both were independently associated with outcome, with a higher rate of change associated with increased risk of failure.

**Conclusion:**

Temporal changes in serum sodium during EVD wean attempts after subarachnoid hemorrhage may represent a new trigger for post-SAH dysnatremia. These changes are not directly explained by iatrogenic exposures. These hypothesis-generating findings warrant validation in prospective trials.

## 1 Introduction

Changes in serum sodium are common following non-traumatic subarachnoid hemorrhage (SAH) (1). Sodium is actively managed during the intensive care unit (ICU) care of these patients by using osmotherapy to induce hypernatremia or correcting hyponatremia. Hyponatremia, which is more common than hypernatremia in this patient population (excluding iatrogenic or induced hypernatremia), occurs due to the syndrome of inappropriate antidiuretic hormone secretion (SIADH) or cerebral salt wasting syndrome (CSWS) (1–3). In both, inappropriate natriuresis occurs, leading to a decrease in serum sodium. While in CSWS the intravascular volume reduces in response, SIADH is considered normo-volemic (2). However, this distinction is difficult to adjudicate at the bedside, and the treatment response (adjusting sodium and volume) is often similar in the acute, ICU stage of SAH treatment (3–5).

The timing of the decrease in sodium has been recorded in two main phases: hyper-acute (first 3 days) and delayed (days 7–10) (6). In addition, hyponatremia precedes cerebral vasospasm based on several retrospective cohorts (7–10) and a meta-analysis (11). Despite this association with vasospasm, or the need for endovascular interventions, hyponatremia was not found to be associated to long term clinical outcomes (11).

Another common complication following SAH is obstructive hydrocephalus. Hydrocephalus is commonly treated with an external ventricular drain (EVD) for cerebrospinal fluid (CSF) diversion during the acute phase (5). Following EVD placement, many centers, including the institution where this study was conducted, leave the EVD open for drainage for several days, followed by a gradual wean in which the EVD chamber is periodically raised acting as an increased pressure valve that challenges the internal CSF circulation (12). For example, at our center the EVD is kept open until after the vasospasm period has resolved (usually about 7 days if no vasospasm is detected, and longer if vasospasm is detected until it is treated and resolved). When a wean trial begins, the EVD chamber is elevated by 5 cm per day until it reaches 20 cm, relative to the tragus. If there is no clinical concern for worsening hydrocephalus, such as elevated intracranial pressure (ICP), the EVD is clamped for 24 h. At that time, a CT scan is obtained for additional information to supplement the clinical exam and an ICP measurement is taken to determine if the EVD can be removed. Another approach maintains the EVD closed apart from times in which the intracranial pressure (ICP) is elevated, followed by a rapid removal of the drain (13). Regardless of the approach, the outcome of a failed wean (for example, due to worse hydrocephalus on imaging) could be a subsequent attempt several days after, or the placement of a permanent ventriculoperitoneal shunt (VPS) for ongoing CSF diversion. In our unit, most patients will have 2–3 failed attempts prior to the determination that a VPS is required. In a recent meta-analysis, several risk factors and predictors for EVD wean failure and need for a VPS were identified, including high clinical grade, blood burden from the initial hemorrhage and age (14). This understanding led to the development of several predictive models aimed to identify wean failure, and likelihood of shunt dependence early on, achieving an area under the curve (AUC) of the receiver operating characteristic (ROC) of 0.76–0.88 (15–17).

At the study institution, the EVD is also used for intrathecal nicardipine treatment to address cerebral vasospasm to prevent delayed cerebral ischemia (18). Therefore, the EVD is usually weaned after vasospasm has resolved. We observed that some patients who cannot tolerate the EVD wean demonstrate a decrease in their serum sodium concentrations, a relationship that, to our knowledge, has not been previously described. Herein, we aim to describe this relationship by performing a retrospective analysis of a large single-center cohort of patients with SAH who required an EVD placement. The study hypothesized that a decrease in serum sodium during the EVD wean process will be associated with a failed wean attempt.

## 2 Methods

In this retrospective analysis, all adult patients admitted to the neuroscience ICU at Emory University Hospital (Atlanta, GA, USA) with a diagnosis of non-traumatic SAH between 2012 and 2022 were retrospectively screened. Adult (>18 years) patients who required an EVD as part of their standard care were included in this analysis. Patients were excluded if they had an EVD for fewer than 4 days, had two or fewer sodium measurements during the wean attempt, did not survive the admission or were discharged to hospice, or there was no wean attempt documented.

Hourly measurements were collected from the electronic medical chart for the EVD chamber height, CSF output and ICP. All serum sodium measurements and medications used during the ICU admission were also collected. The outcome of VPS placement was determined from the electronic medical records.

An EVD wean attempt was defined separately for height and clamp attempts. A height wean was identified based on differences in consecutive local minimum and maximum heights of the EVD chamber. Specifically, a height wean attempt required at least a 10 cm difference between a specific patient’s minimum and maximum. See Supplementary Figure S1 for an example. Any missing height observations were assumed to be equal to the last recorded value. A clamp attempt was identified when there was zero CSF output over a ≥ 8 h period. Missing CSF readings were assumed to have zero output. In cases where a clamp attempt began during an ongoing height wean, the original height wean label was retained. In total, there were 421k observations of which 70k, approximately 16%, were missing either a height or CSF value. Standard protocol in the ICU at Emory stops the documentation when the clamp is in place, as no changes are expected. This protocol explains the majority of the missing data.

A successful EVD wean attempt was defined through two criteria: first, it must be the final attempt with no further ICP/CSF output readings; second, there was no subsequent VPS placed. A wean failure was defined as either re-opening and lowering of the EVD chamber (in preparation for another attempt) or if the EVD was replaced by a VPS.

Iatrogenic sodium exposure was quantified by multiplying the volume of intravenous fluid administered times the concentration of sodium. This calculation included all types of sodium containing solution, including isotonic, hypertonic, or carriers for medication infusions, regardless of the indication or route of administration (intravenous or orally/via a feeding tube). The result was measured as total milliequivalent (mEq) sodium. Sodium exposure was reported cumulatively over a period relative to the start of the EVD wean. The beginning of the EVD wean was defined as day 0, and the exposure was measured from day −2 (i.e., 2 days prior to the wean attempt) until the end of the wean trial or day +5, whichever came first.

### 2.1 Statistical methods

Sodium trends were created using a LOWESS model tuned to include 30% of the nearest observations for estimating each point (19). All times were calculated relative to the start of a wean. For the sodium trends, we generate 95% confidence intervals using bootstrapping over 100 iterations. The relationships between probability of a good outcome and the independent variables were modeled using mixed effect logistic regression. We used two methods for selecting the subset of variables to include in the multivariable logistic regression. First, we used a stepwise selection method which iteratively adds or subtracts a variable depending on the impact to AIC. Second, we exhaustively evaluated all combinations of variables while selecting based on AIC. Statistical software used were Python version 3.9.21 and R version 4.3.2.

Descriptive statistics were calculated as mean ± standard deviation for continuous parameters. We used percentages for the categorical variables along with the median, lower, and upper quantiles. Odds ratios were calculated using a step size of 25 milliliter for the cumulative CSF output and a step size of 1 for all other values. Hypothesis testing for the three demographic categories included one-way ANOVA tests for the continuous variables, and chi-square tests for the proportions and categorical data. The *p*-values derived from the mixed effect logistic regression models were calculated using a Wald test with the null hypothesis that the coefficient equals zero and are denoted with [w]. We use AUC to evaluate the predictive performance of our final model averaged over 100 random splits of 90% training data and 10% test data.

## 3 Results

### 3.1 Patient cohort

**Figure 1:**
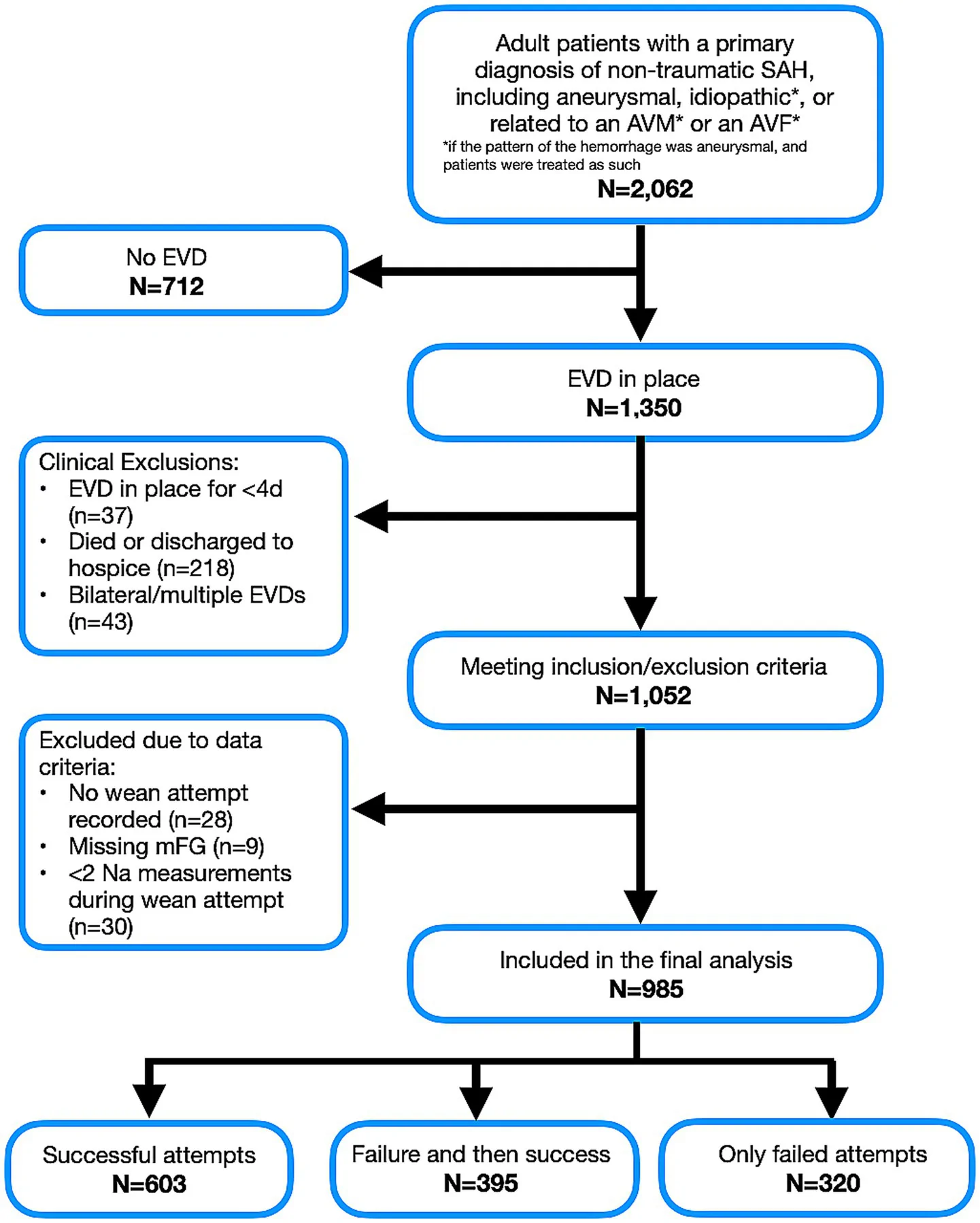
Flowchart for the patient cohort.

There were 2,062 patients admitted with non-traumatic SAH between 2012 and 2022. Patients were excluded per the criteria listed above, and as detailed in Figure 1. The remaining 985 patients underwent 1,318 unique wean attempts, of which 790 (59.9%) were successful and 528 were unsuccessful (Figure 1). A total of 195 patients required a VPS. The entire patient cohort, and the cohort separated by outcome is detailed in Table 1.

**Table 1:** Baseline patient demographics, clinical and radiographic severity, hemorrhage etiology, and treatment.

Characteristic	All	Success	Failure	Mixed	*p*-value
*N* (patients)	985	603	195	187	
Age (mean ± SD)	53.6 ± 12.5	53.2 ± 13.0	55.1 ± 12.1	53.4 ± 11.0	0.21
Sex (female, *n* [%])	300 (30.5%)	186 (30.8%)	58 (29.7%)	56 (29.9%)	0.95
Race/ethnicity					0.05
White	360 (37%)	229 (38.0%)	74 (37.9%)	57 (30.4%)	
African American	410 (42%)	246 (40.8%)	75 (38.5%)	89 (47.5%)	
Asian	32 (3%)	14 (2.3%)	13 (6.6%)	5 (2.7%)	
Unknown/other	166 (17%)	101 (16.7%)	32 (16.4%)	33 (17.6%)	
Hispanic ethnicity	17 (2%)	13 (2.1%)	1 (0.5%)	3 (1.6%)	
WFNS (median [Q1–Q3])	2 [1–4]	2 [1–3]	4 [2–4]	2 [1–4]	<0.01
1	268 (27%)	191 (31.7%)	26 (13.3%)	51 (27.3%)	
2	354 (36%)	233 (38.6%)	47 (24.1%)	74 (39.6%)	
3	65 (7%)	36 (6.0%)	18 (9.2%)	11 (5.9%)	
4	242 (25%)	118 (19.6%)	89 (45.6%)	35 (18.7%)	
5	56 (6%)	25 (4.1%)	15 (7.7%)	16 (8.6%)	
mFG (median [Q1–Q3])	4 [3–4]	4 [3–4]	4 [4–4]	4 [4–4]	<0.01
0	3 (0.3%)	3 (0.5%)	0 (0.0%)	0 (0.0%)	
1	42 (4%)	32 (5.3%)	4 (2.1%)	6 (3.2%)	
2	73 (7%)	57 (9.5%)	11 (5.6%)	5 (2.7%)	
3	139 (14%)	99 (16.4%)	10 (5.1%)	30 (16.0%)	
4	728 (74%)	412 (68.3%)	170 (87.1%)	146 (78.1%)	
Etiology					<0.01
Aneurysmal	834 (85%)	481 (79.8%)	180 (92.3%)	173 (92.5%)	
Idiopathic (angio-negative)	130 (13%)	109 (18.1%)	12 (6.2%)	9 (4.9%)	
Other (e.g., AVM, AVF)	21 (2%)	13 (2.1%)	3 (1.5%)	5 (2.7%)	
Initial treatment					<0.01
Craniotomy	241 (24%)	145 (24.0%)	56 (28.7%)	40 (21.4%)	
Endovascular	602 (61%)	339 (56.2%)	126 (64.6%)	137 (73.2%)	
No treatment	13 (1%)	9 (1.5%)	1 (0.5%)	3 (1.5%)	
Angio-negative	129 (13%)	108 (17.9%)	12 (6.2%)	9 (4.7%)	
Time to wean attempt (days)	10.6 ± 6.1	8.5 ± 4.1	12.1 ± 6.5	11.8 ± 4.1	<0.01
Wean attempt (median [Q1–Q3])	1 [1–2]	1 [1–1]	2 [2–3]	2 [2–2]	<0.01
1	698 (70%)	603 (100%)	95 (48.7%)	0 (0.0%)	
2	246 (25%)	0 (0.0%)	77 (39.5%)	169 (90.3%)	
3	36 (4%)	0 (0.0%)	21 (10.8%)	15 (8.0%)	
4	5 (1%)	0 (0.0%)	2 (1.0%)	3 (1.6%)	
Ventriculoperitoneal shunt	195 (24.6%)	0	195 (100%)	0	<0.01

The cohort is detailed as a whole, and by patient outcome. The success group had one successful attempt. The failure group describes those who had only failed attempts. The “Mixed” group had initial failures but an ultimate success. *p*-values represent the comparison between three patient stratifications. WFNS, World Federation of Neurological Surgeons; mFG, Modified Fisher Grade; AVM, Arteriovenous malformation; AVF, Arteriovenous fistula.

### 3.2 Changes in serum sodium and wean outcome

**Figure 2:**
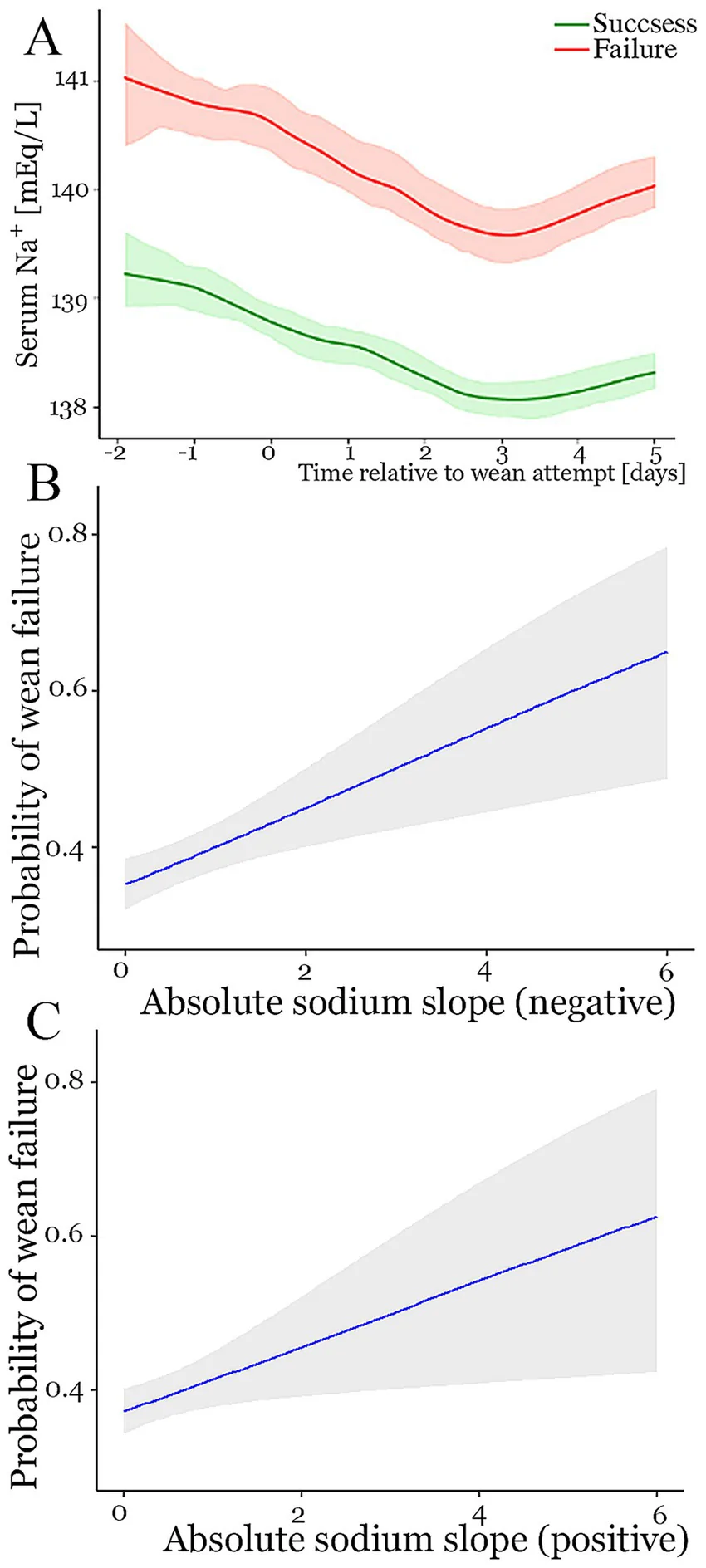
The relationship between serum sodium over time and risk of EVD wean failure. **(A)** Change in average serum sodium concentration over time in relationship to the wean initiation (time 0). A clear difference appears prior to and throughout the wean attempt with a steeper decrease in sodium in the group that failed the wean attempt. (Shaded area—95% confidence interval). **(B)** Probability of successful EVD wean in relation to a decrease in serum sodium concentration during the time frame starting from 48 h before the wean to 72 h after for patients who demonstrated a decrease with units of mEq/L/day. Note the slope is represented as an absolute value, and therefore positive, despite describing a decrease in serum sodium. **(C)** Probability of successful EVD wean in relation to the increase in serum sodium concentration during the time frame starting from 48 h before the wean to 72 h after, for patients demonstrating an increase. EVD, external ventricular drain; Na^+^, sodium; mEq/L, milliequivalent per liter.

Upon comparing the successful and failed attempt trials, we found that the initial sodium concentration (mEq/L) in these two groups was 139.3 ± 8.5 vs. 140.7 ± 6.6 (*p* < 0.001 [w]) on the day of wean initiation. Even at 2 days prior to the wean initiation, this difference was present (139.4 ± 8.0 vs. 140.8 ± 6.8, *p* < 0.001 [w]). The analysis demonstrated a decrease of 0.8 ± 7.8 mEq/L of serum sodium in the group with a successful wean during the first 3 days of the wean attempt, versus 1.5 ± 5.8 in the group that failed the wean (*p* = 0.048 [w]), before reaching a plateau. This change was most prominent in the first 2 days of the wean attempt (Figure 2A).

However, the directionality of sodium changes was heterogenous. Approximately 58% of weaning trials were associated with a decrease in serum Na^+^, compared to 42% with an increase. Nevertheless, the slope of the absolute change in sodium had a positive association with the risk of wean failure (OR [95% CI] of 1.324 [1.174–1.506]) in either direction. When analyzing patients who had a decrease (Figure 2B) or an increase (Figure 2C) in Na^+^ separately, a similar trend appears: a larger change in serum Na^+^ was associated with a higher risk of wean failure. Supplementary Figure S2 shows the distribution of the positive and negative slope values across all wean attempts.

### 3.3 Analysis of possible confounders

To examine if the difference in the sodium changes was iatrogenic, we first measured each patient’s total sodium exposure to any type of pharmaceutical intervention (either PO or IV) as total milliequivalents (mEq) of sodium. There was no statistically significant difference in the cumulative sodium intake with medications during the wean attempt between the success and failure groups regardless of the time window evaluated (Table 2). A second potential confounder is fludrocortisone, a mineralocorticoid often used to address hyponatremia in patients with SAH. The exposure to fludrocortisone at any dose was more common in the group with a successful wean (45.1% vs. 39.2%, *p* < 0.001 [w]). After splitting the cohort between those who either demonstrated a decrease in sodium (Supplementary Table S1) or an increase (Supplementary Table S2) during the wean attempt, similar trends appear. In this subgroup analysis, only the exposure to fludrocortisone in the decreasing sodium group remained statistically significant with 52.7 and 37.6% in the successful and failure groups, respectively (*p* < 0.001 [w]). Additionally, we analyzed exposure to vasopressin as this can reduce serum sodium levels. The difference in proportion of patients exposed to vasopressin was statistically significant at 2.3% and 5.9% (*p* = 0.020 [w]) for the success and failure classes, respectively.

**Table 2:** Iatrogenic exposures that could change serum sodium during the wean attempt.

Variable	Success (*n* = 790)	Failure (*n* = 528)	*p*-value
Cumulative sodium (−2 to 1) [mEq]	26.9 ± 63.4	23.6 ± 35.8	0.676
Cumulative sodium (−2 to 3)	32.6 ± 88.2	28.9 ± 47.8	0.275
Cumulative sodium (−2 to 5)	35.4 ± 105.7	30.7 ± 51.7	0.273
Exposure to fludrocortisone during the wean attempt	45.4% (359)	35.2% (186)	<0.001
Exposure to vasopressin during the wean attempt	2.3% (18)	4.5% (24)	0.020

Cumulative sodium is the sum of all sodium delivered as a medication in all documented routes (primarily intravenous and enterally). This analysis was performed at different time frames relative to the start of the wean attempt. For example, values in parentheses indicate the window of analysis in days where (−2 to 1) indicates a 72-h period from 2 days prior to the wean start to 1 day after the start. Exposure to Fludrocortisone or vasopressin were represented as separate binary variables, regardless of the dose or specific indication. The *p*-values were calculated using a Wald test for a mixed effect regression coefficient.

### 3.4 Risk factors for EVD wean failure

**Figure 3:**
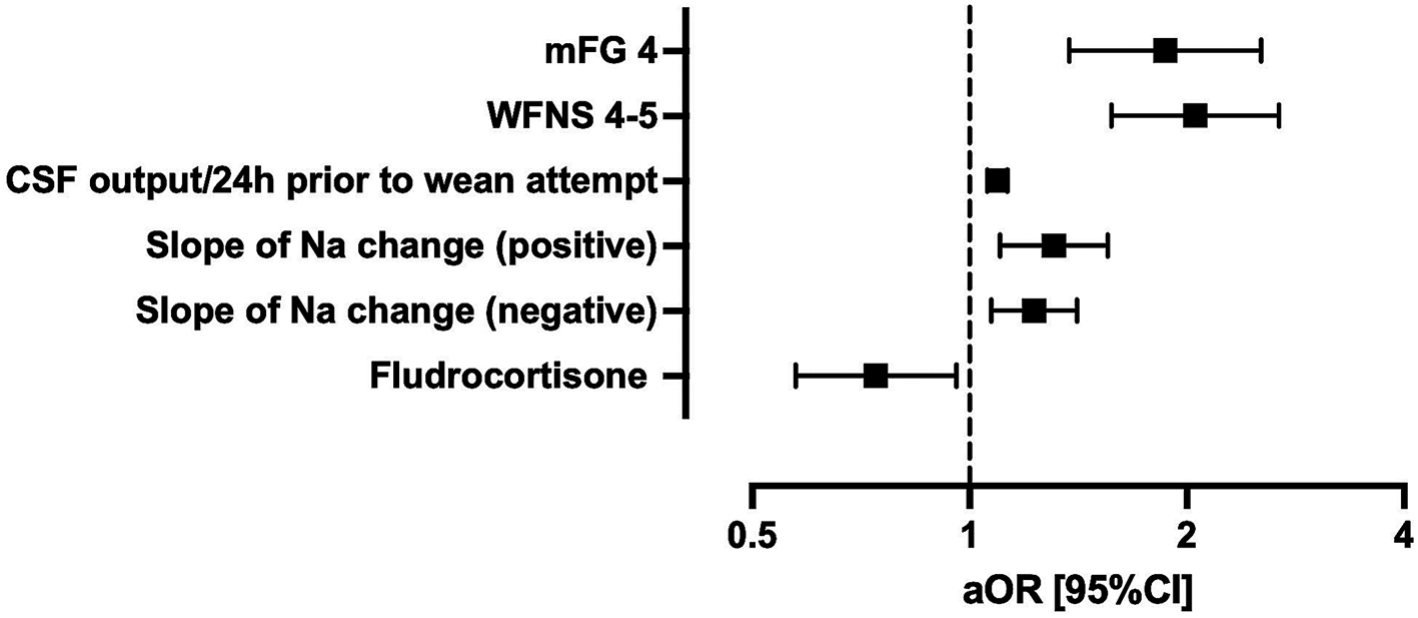
Multivariable analysis to predict wean failure. Results are presented as adjusted odds ratio (aOR) with 95% confidence interval (CI). mFG, Modified Fisher Grade; WFNS, World Federation of Neurological Surgeons. Slope of Na change refers to the change over time (mEq/L/day) in serum sodium starting from 2 days before the wean attempt to 3 days after and separated into attempts in which the change was overall positive or negative. Fludrocortisone refers to exposure at any dose or indication.

Next, we performed univariate mixed effect logistic regression analyses with commonly used predictors for EVD wean fail along with our current findings (Supplementary Table S3). Parameters that demonstrated statistically significant associations were input into the multivariable analysis with variable selection with the results shown in Figure 3. The slope of change in sodium during the EVD wean attempt was found to be independently associated with wean failure (OR [95% CI] of 1.262 [1.110–1.436]). When split into separate variables for positive and negative slopes, the effect was similar. Interestingly, exposure to fludrocortisone was independently associated with lower failure rates (OR [95% CI] of 0.74 [0.57–0.96]). The AUC of our final model averaged 0.662 over 100 random splits of our data.

## 4 Discussion

In this retrospective analysis of a large, single center cohort of patients with non-traumatic SAH complicated by obstructive hydrocephalus, we identified a potential novel predictor of EVD weaning trial failure. The change in serum sodium during the days in which the EVD chamber was weaned and eventually clamped was independently associated with the risk of wean failure. Although 58% of recorded events showed a decrease in sodium, counter to our initial hypothesis, an increase in sodium was also independently associated with wean failure. We analyzed the main potential iatrogenic confounders and showed that it is unlikely that the change in sodium is related to the medical administration of sodium. In contrast, we found that the use of fludrocortisone, usually used to treat hyponatremia in CSWS, was associated with a lower risk of failure. These hypothesis-generating data expand our understanding of dysnatremia in the settings of SAH and perhaps help develop new biomarkers to identify non-resolving obstructive hydrocephalus post SAH.

A correlation between hydrocephalus and hyponatremia was previously reported in a pediatric population (20). This was generally attributed to the syndrome of inappropriate antidiuretic hormone secretion (SIADH). A possible anatomic-pathophysiological explanation could lay in the presence of nuclei related to sodium and water hemostasis in close proximity to the ventricular system, such as the subfornical organ which is adjacent to the third ventricle wall (21). However, a similar correlation was not reported in adults, and specifically not in SAH. One report suggested no correlation between sodium and hydrocephalus in a small (*n* = 67) retrospective analysis of mixed neurosurgical patient population (22).

When considering the specific setting of EVD weaning, this analysis reinforces that certain patient parameters are associated with the risk of a wean failure. Having a high clinical grade (WFNS 4 or 5), radiographic grade (mFG 4) and high CSF output have all previously been shown to predict wean failure (23, 24). To our knowledge, this is the first study to show that, on average, the sodium level decreases throughout the EVD wean process. When comparing the serum sodium at the beginning of the EVD wean trial between successful and unsuccessful weans, a small, but statistically significant difference was observed. However, this initial difference was not independently predictive of the outcome. Similarly, the difference between maximum and minimum sodium measurements was not associated with outcome. The change over time, described by the slope, was associated with the wean outcome, with larger slopes associated with higher risk for wean failure.

The combination of the timing of the wean attempt and our clinical practice mitigates potential confounders in this cohort. Weans occurred on average on day 10, which indicates that patients were generally past their worst cerebral edema phase, and were unlikely to be treated with osmotherapy at that time (25). Indeed, differences in exposure to sodium in the days immediately preceding and during the wean trial was negligeable (Table 2). For reference, a common liter of crystalloid infusion contains 140–154 mEq of sodium, while a common hypertonic saline solution used in our unit contains 120–140 mEq of sodium per dose (26). During the wean attempt patients received on average 20–40 mEq of sodium over several days. Furthermore, the change in sodium is unlikely to be driven by cerebral vasospasm, a known cause for hyponatremia post SAH (7–10). At our unit, we utilize the EVD for intrathecal nicardipine as a common treatment for cerebral vasospasm whenever an EVD is present (18), and the EVD wean is delayed until the vasospasm is resolving. Therefore, in this cohort, the wean trial is unlikely to be confounded by radiographic or clinical vasospasm.

The change in serum sodium observed in this analysis is small in absolute terms. The clinical laboratory measurements of sodium are reported as integers over a relatively narrow range making these findings difficult to support developing a new independent biomarker for EVD wean failure. Therefore, these results cannot directly impact clinical practice. However, these results could be investigated further in future studies. First, the change in serum sodium could be added to other established clinical predictors to develop a new multivariable model. Second, these findings could elucidate an often challenging diagnosis related to SAH, namely cerebral salt wasting syndrome (27). Most patients who failed their weaning had a concomitant decrease in sodium. Additionally, the treatment with fludrocortisone, a common intervention to address cerebral salt wasting, was shown to be associated with a higher success rate of EVD weaning. It is also interesting to note that vasopressin, a pressor also used to address diabetes insipidus (DI) (28) and decreases sodium concentration, was more common in the group that failed the EVD wean. However, vasopressin use was not independently associated with the wean result. It is worth stressing that none of the patients in this cohort suffered from DI related to cerebrovascular arrest, since all patients who did not survive the admission were excluded from the analysis. While we cannot exclude the possibility that some of the patients were treated for DI, the far more common use of vasopressin is as a vasoactive drip in the settings of distributive shock. Third, the fact that an increase in sodium, contrary to our initial hypothesis, was also associated with wean failure may suggest a broader link between hydrocephalus and sodium regulation. For example, there could be a mirror syndrome to CSWS, which could be confounded by hyperosmolar therapy in SAH. Given the established correlation between hyponatremia and complications, the clinical focus is on the lower end rather than on patients who develop a late elevation in serum sodium (11). These findings align with recent evidence associating overall sodium fluctuations with SAH-related complications and outcomes (29).

Sodium is a highly regulated electrolyte, with little variability under normal health conditions (21). The fact that a change in sodium in either direction is related with a wean failure may indicate a loss of that regulation. As described by Godin and Buchman, critical illness is characterized by a loss of biological oscillators that keep all organ functions synchronized (30). The result is an increase in variability, which is another way to describe the change on sodium over just a few days. In our cohort, the risk of wean failure increased with the magnitude of sodium change. A loss of regulation that leads to a greater change in serum sodium suggests a pathologic state of persistent hydrocephalus. Yet, this interaction is complex, since there are many physiologic and pathologic confounders starting from the normal regulation of sodium, to continue with iatrogenic interventions, as mentioned, fluid balance, possible endocrine dysregulation resulting in CSWS or SIADH, acute kidney injury etc. The exact mechanism that links worsening hydrocephalus with changes in sodium requires further investigation.

This study has several limitations. First, the retrospective, uncontrolled, single center design for a single disease limits the generalization of the results. Second, the potential iatrogenic confounders tested are interventions that are not well protocolized. For example, the threshold to initiate, dose, and the propensity to continue fludrocortisone was physician dependent. Similarly, the decisions to wean off hyperosmolar therapy and to start the EVD wean are non-protocolized and vary between clinicians. This limitation is addressed, to some extent, by the large size of the dataset analyzed. However, the granularity of this dataset does not allow us to identify if some patients had a decrease in sodium as a return to a normal value since hyperosmolar therapy was stopped 4–5 days prior; and on the contrary, an increase can otherwise occur, for example, when patients remain fasting for procedures. Third, it is unclear if the wean outcome is related to the manner by which the EVD was weaned, since there is some evidence to support a faster wean process compared with our center’s usual practice (31). Fourth, the resolution of the available data prevents a more detailed analysis of the relationship between the intensity and duration of osmotherapy and other sodium-related interventions, possible fluid shifts, neuro-hormonal regulation or other confounders prior to the wean attempt, which may explain the changes in sodium for some of the patients. In that sense, disease severity and related complications (for example, acute kidney injury) could influence the sodium concentration. Given the high variability in the disease process and in clinical practice responding to it, one can only control for all possible interventions with a well-controlled prospective trial. Regardless, this observation remains novel and hypothesis generating, and one that will require future research.

To conclude, post SAH temporal changes in serum sodium during an EVD wean attempt emerge as a possible biomarker to predict wean outcome. Although decreases in sodium were more common, both increases and decreases during the wean trial were found to be independently associated with wean failure and cannot be directly explained by medication administered in this retrospective single center analysis. This hypothesis-generating data can guide future hydrocephalus research and support the understanding of dysnatremia, hydrocephalus and related treatments.

## Data Availability

The raw data supporting the conclusions of this article will be made available by the authors, upon reasonable request.
