# Supplementary material for: Changes in serum sodium during an external ventricular drain weaning in patients with subarachnoid hemorrhage
Source: Front Neurol. 2026 Aug 19;17:1843156. doi: 10.3389/fneur.2026.1843156 (PMC13533758; doi:10.3389/fneur.2026.1843156)
Supplement: Supplementary file 1 [file Supplementary_File_1.docx]

**Supplementary information for: Changes in serum sodium during an external ventricular drain weaning in patients with subarachnoid hemorrhage – a single center retrospective analysis**

**Figure S1**: Example height charts with the start and end for the wean attempt indicated by the grey and black dashed lines, respectively. In this case the initial attempt failed, and a repeat one was completed a few days following the first one.

**Figure S2**: Distribution of the positive and negative slope values across all wean attempts.

**Table S1**: Iatrogenic exposures that could change serum sodium during the wean attempt in patients who demonstrated an increase in sodium during the weaning trial. Cumulative sodium is the sum of all sodium delivered as a medication in all documented routes (primarily intravenous and per os). This analysis was performed at different time frames relative to the start of the wean attempt. For example, values in the parenthesis indicate the window of analysis in days where (-2 to 1) indicates a 72-hour period from two days prior to the wean start to 1 day after the start. Exposure to Fludrocortisone or vasopressin were represented as separate binary variables, regardless of the dose or specific indication.

| **Slope Pos (n = 553)** | **Success**  **(n=332)** | **Failure**  **(n=221)** | **p-value** |
| --- | --- | --- | --- |
| **Cumulative Sodium (-2 to 1) [mEq]** | 14.7±27.4 | 17.9±32.0 | 0.526 ** |
| **Cumulative Sodium (-2 to 3)** | 18.5±36.3 | 29.0±60.1 | 0.131 ** |
| **Cumulative Sodium (-2 to 5)** | 18.3±35.9 | 29.1±63.8 | 0.148 ** |
| **Exposure to Fludrocortisone during the wean attempt** | 52.7% (175) | 37.6% (83) | <0.001 ** |
| **Exposure to Vasopressin during the wean attempt** | 2.1% (7) | 4.5% (10) | 0.110 ** |

**Table S2**: Iatrogenic exposures that could change serum sodium during the wean attempt in patients who demonstrated a decrease in sodium during the weaning trial. Cumulative sodium is the sum of all sodium delivered as a medication in all documented routes (primarily intravenous and per os). This analysis was performed at different time frames relative to the start of the wean attempt. For example, values in the parenthesis indicate the window of analysis in days where (-2 to 1) indicates a 72-hour period from two days prior to the wean start to 1 day after the start. Exposure to Fludrocortisone or vasopressin were represented as separate binary variables, regardless of the dose or specific indication.

| **Slope Neg (n=765)** | **Success**  **(n=458)** | **Failure**  **(n=307)** | **p-value** |
| --- | --- | --- | --- |
| **Cumulative Sodium (-2 to 1) [mEq]** | 37.1±80.9 | 27.1±37.7 | 0.440 ** |
| **Cumulative Sodium (-2 to 3)** | 43.8±112.5 | 28.8±39.2 | 0.406  ** |
| **Cumulative Sodium (-2 to 5)** | 49.3±137.3 | 31.6±43.6 | 0.379 ** |
| **Exposure to Fludrocortisone during the wean attempt** | 40.1% (184) | 33.5% (103) | 0.076 ** |
| **Exposure to Vasopressin during the wean attempt** | 2.4% (11) | 4.6% (14) | 0.103  ** |

**Table S3**: Univariate regression analysis to predict wean failure. Results are presented as odds ratio (OR) with 95% confidence interval (CI). mFG – modified Fisher Grade, WFNS - World Federation of Neurological Surgeons. Slope of Na change refers to the change over time in serum sodium starting from 2 days before the wean attempt to 3 days after and separated into attempts in which the change was overall positive or negative. Vasopressin and Fludrocortisone refer to any exposure at any dose or indication during the same time frame.

The parameters were defined as following:

- Delta Na – The difference (absolute value) in sodium for the maximum and minimum values during a wean attempt if the maximum occurred prior to the minimum, zero if not (mg/dL)
- SlopeABS – The magnitude (absolute value) for the slope of the least squares regression line for sodium measurements 48 hours prior to the wean start until 72 hours after the wean start. (mg/dL/hr)
- SlopeNeg – The magnitude (absolute value) for the slope of the least squares regression line for sodium measurements 48 hours prior to the wean start until 72 hours after the wean start. (mg/dL/hr)
- SlopePos – The magnitude (absolute value) for the negative slope of the least squares regression line for sodium measurements 48 hours prior to the wean start until 72 hours after the wean start (mg/dL/hr). This value is zero if the slope was negative.
- Na 48h prior – The first sodium measurement less than 48 hours prior to the start of the wean (mg/dL)
- Age – Patient age (years)
- CSF 24h prior – The cumulative CSF measurements less than 24 hours prior to the start of the wean attempt (mL).
- WFNS 4-5 – Binary indication of whether the patient had a WFNS grade of 4 or 5 (1-yes, 0-no).
- mFG 4 – Binary indication of whether the patient had a Fisher grade of 4 (1-yes, 0-no).
- Vassopressin – Binary indication of whether the patient received vasopressin within 72 hours of the start of the wean or at any time during the wean attempt (1-yes, 0-no).
- Fludrocortisone - Binary indication of whether the patient received fludrocortisone within 72 hours of the start of the wean or at any time during the wean attempt (1-yes, 0-no)

| **Variable** | **OR** | **Lower** | **Upper** | **P-val** |
| --- | --- | --- | --- | --- |
| Delta Na | 1.013 | 0.991 | 1.035 | 0.24 |
| SlopeABS | 1.324 | 1.174 | 1.506 | 0.00 |
| SlopeNeg | 1.226 | 1.081 | 1.399 | 0.00 |
| SlopePos | 1.188 | 1.035 | 1.386 | 0.02 |
| Na 48h prior | 1.055 | 1.030 | 1.083 | 0.00 |
| Age | 1.009 | 0.997 | 1.019 | 0.06 |
| CSF 24h prior | 1.077 | 1.044 | 1.115 | 0.00 |
| WFNS 4-5 | 2.458 | 1.916 | 3.195 | 0.00 |
| mFG 4 | 2.218 | 1.663 | 3.002 | 0.00 |
| Vasopressin | 2.203 | 1.143 | 4.371 | 0.02 |
| Fludrocortisone | 0.639 | 0.499 | 0.811 | 0.00 |
| Total Na [mEq] | 0.999 | 0.996 | 1.002 | 0.43 |

- Total Na [mEq] - The total amount of sodium converted to milliequivalent administered to a patient within 48 hours of the start of the wean to 72 hours after it began.
